# Respiratory virus infections of the lower respiratory tract elevate bronchoalveolar lavage eosinophil fraction: a clinical retrospective study and case review

**DOI:** 10.1186/s12890-023-02402-x

**Published:** 2023-04-06

**Authors:** Daijiro Nabeya, Michika Setoguchi, Shiho Ueno, Takeshi Kinjo

**Affiliations:** Department of Infectious, Respiratory and Digestive Medicine, Graduate School of Medicine, University of the Ryukyus, 207 Uehara, Nishihara, Okinawa 903-0215 Japan

**Keywords:** Community-acquired respiratory virus, Eosinophilic airway inflammation, Eosinophils, Bronchoalveolar lavage, Eosinophilic pneumonia

## Abstract

**Background:**

Eosinophilic airway inflammation caused by respiratory virus infection has been demonstrated in basic research; however, clinical investigations are lacking. To clarify the extent to which respiratory virus infection induces airway eosinophilic inflammation, we reviewed the results of bronchoalveolar lavage (BAL) and respiratory virus testing performed at our hospital.

**Methods:**

Among the BAL procedures performed at the University of the Ryukyu Hospital from August 2012 to September 2016, we collected cases of acute respiratory disease in which multiplex polymerase chain reaction (PCR) was used to search for respiratory viruses. The effect of respiratory virus detection on BAL eosinophil fraction was analyzed using statistical analysis. A case study was conducted on respiratory virus detection, which showed an elevated BAL eosinophil fraction.

**Results:**

A total of 95 cases were included in this study, of which 17 were PCR-positive. The most common respiratory virus detected was parainfluenza virus (eight cases). The PCR-positive group showed a higher BAL eosinophil fraction than the PCR-negative group (p = 0.030), and more cases had a BAL eosinophil fraction > 3% (p = 0.017). Multivariate analysis revealed that being PCR-positive was significantly associated with BAL eosinophil fraction > 1% and > 3%. There were nine PCR-positive cases with a BAL eosinophil fraction > 1%, of which two cases with parainfluenza virus infection had a marked elevation of BAL eosinophil fraction and were diagnosed with eosinophilic pneumonia.

**Conclusions:**

Cases of viral infection of the lower respiratory tract showed an elevated BAL eosinophil fraction. The increase in eosinophil fraction due to respiratory virus infection was generally mild, whereas some cases showed marked elevation and were diagnosed with eosinophilic pneumonia. Respiratory virus infection is not a rare cause of elevated BAL eosinophil fraction and should be listed as a differential disease in the practice of eosinophilic pneumonia.

## Background

Respiratory viruses are often implicated in the exacerbation of respiratory diseases, including asthma exacerbations, which is a well-known fact [1]. This is thought to be due to the eosinophilic inflammation in the lower respiratory tract caused by respiratory virus infection. Basic medical studies have shown that respiratory viruses induce eosinophilic inflammation in the airways [2]. However, clinical studies are scarce and insufficient to determine the extent of eosinophilic inflammation of the lower respiratory tract caused by viral respiratory infections. To confirm this, clinical specimens of the lower respiratory tract of infected patients are required.

In our institution, when bronchoalveolar lavage (BAL) fluid is collected in clinical practice, multiplex polymerase chain reaction (PCR) testing for respiratory viruses is performed as a routine test to differentiate between infectious. Therefore, PCR results and BAL cell fraction can be collected from past data. We retrospectively summarized this data in patients with acute lung disease and investigate whether the detection of respiratory viruses in BAL fluid is associated with increased BAL eosinophil fraction. Additionally, we reviewed cases of respiratory virus infection and elevated BAL eosinophil fraction.

## Methods

We reviewed data from BAL testing and respiratory virus search multiplex PCR testing of BAL fluid at the University of the Ryukyus Hospital from August 2012 to September 2016. To collect cases of acute respiratory virus infections, cases without respiratory symptoms or new lung lesions within two weeks prior to BAL testing were excluded. Multiplex PCR was performed using commercially available kits (Seeplex RV15 OneStep ACE Detection, Seegene, Korea), which can detect multiple community-acquired respiratory viruses, such as adenovirus; coronavirus 229E/NL63; OC43; parainfluenza virus 1, 2, 3, 4, rhinovirus A/B/C; respiratory syncytial viruses A and B; metapneumovirus; enterovirus; influenza viruses A and B; and bocavirus 1/2/3/4. We defined elevated BAL eosinophil fraction as a value greater than 1% [3].

Factors that increased BAL eosinophil fraction were statistically analyzed. Statistical analysis was performed using IBM SPSS Statistics for Windows, version 22.0 (Armonk, NY, USA). Differences among patients’ backgrounds and laboratory test results were compared between the PCR-positive group and the PCR-negative group using the chi-square and Mann–Whitney U tests. Because the PCR-positive group included cases with marked elevation of BAL eosinophil fraction, cutoff values for eosinophil fraction (> 1%, > 3%, and > 5%) were set and analyzed with binary variables as well. Stepwise logistic regression analysis (increasing variables method) was performed to explore factors involved in increased BAL eosinophil fraction. The independent variables were PCR-positive in BAL, user of systemic corticosteroid, user of inhaled corticosteroid, bronchial asthma, history of allergies or allergic reactions, and eosinophils in blood > 500/µL. Independent variables were selected based on the results of the univariate analysis and the clinical importance of airway eosinophilic inflammation.

This study was approved by the Clinical Research Ethics Committee of the University of the Ryukyus.

## Results

**Fig. 1:**
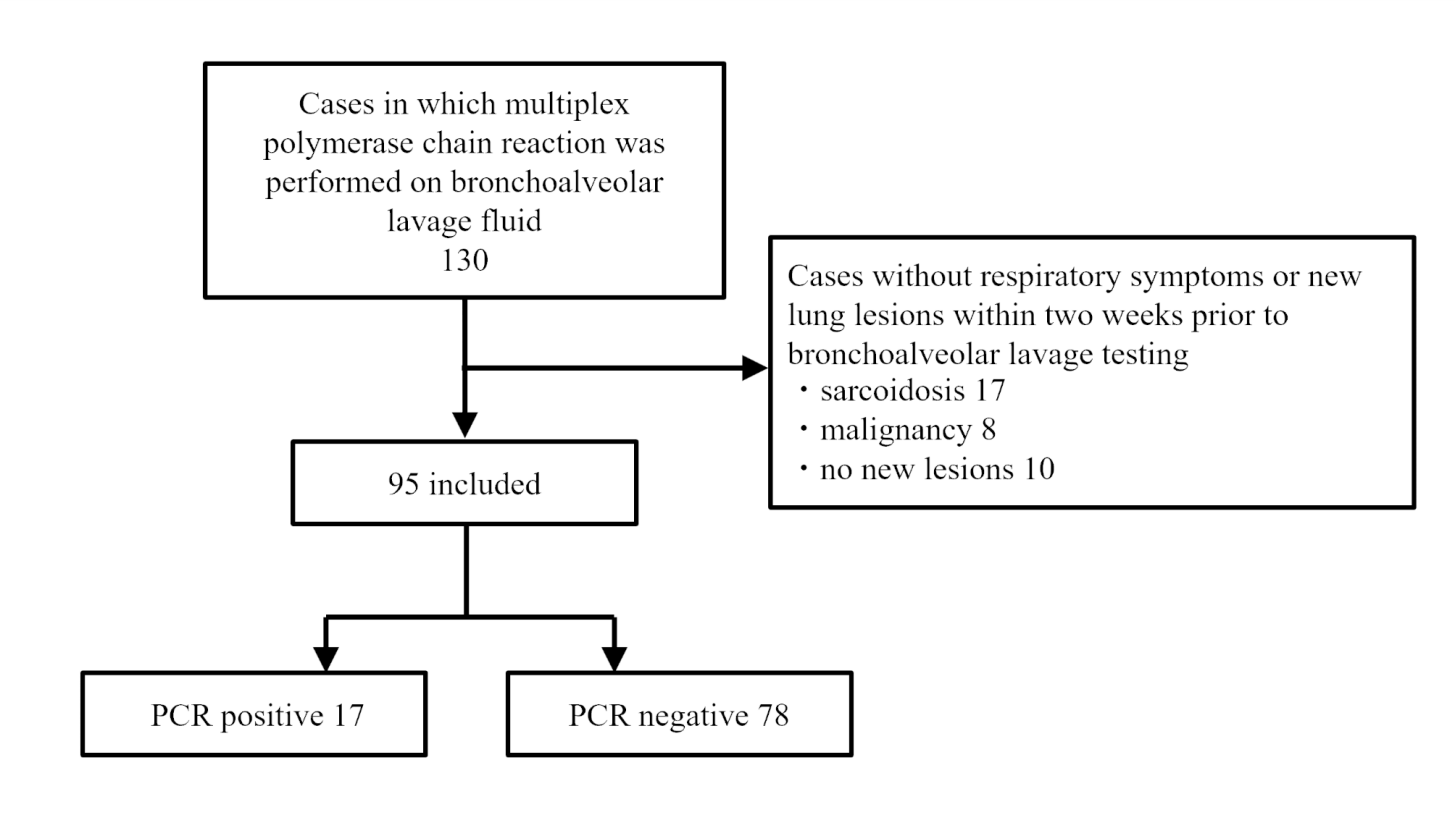
Flow diagram of this study A total of 130 patients with respiratory disease underwent bronchoalveolar lavage and multiplex polymerase chain reaction. Cases without respiratory symptoms or new lung lesions within two weeks prior to bronchoalveolar lavage testing were excluded, and 95 patients were included

Ninety-five cases were included in this study, of which 17 were PCR-positive (Fig. 1). The 17 PCR-positive cases were classified by virus detection: eight parainfluenza virus, three rhinovirus, two enterovirus, two influenza A virus, one respiratory syncytial virus, and one human metapneumovirus. Table 1 shows the patient background. The PCR-positive group tended to be younger and had a higher prevalence of bronchial asthma. The administration of systemic steroids did not differ between the two groups. Table 2 summarizes the final diagnoses of the respiratory diseases in the included cases. They can be broadly divided into respiratory infection and interstitial lung disease; among the PCR-positive cases. Viral pneumonia was diagnosed in two cases, while respiratory virus infections were considered a comorbidity in the other cases. Except for patients with influenza virus infection, none of the patients were treated with antiviral therapy.

**Table 1:** Demographic data of study subjects

	PCR-positive	PCR-negative	*p*-value
N	17	78	
Male, n (%)	10 (59)	56 (72)	0.293
Age, median (range)	55 (24–49)	66 (15–87)	0.009
History of allergies or allergic reactions, n (%)	8 (47)	14 (18)	0.015
Underlying diseases			
Malignancies, n (%)	7 (41)	32 (41)	0.991
Autoimmune diseases, n (%)	4 (24)	15 (19)	0.456
Acquired immunodeficiency syndrome, n (%)	1 (6)	4 (5)	0.636
Bronchial asthma, n (%)	4 (24)	4 (5)	0.032
Chronic obstructive pulmonary disease, n (%)	2 (12)	9 (12)	0.626
User of inhaled corticosteroids, n (%)	3 (18)	3 (4)	0.068
Under immunosuppressive therapy			
Systemic corticosteroids, n (%)	4 (24)	21 (27)	0.519
non-steroidal immunosuppressive therapy, n (%)	5 (29)	11 (14)	0.123
Anti-cancer drugs, n (%)	4 (24)	20 (26)	0.563

**Table 2:** Final diagnoses of pulmonary diseases

	PCR-positive	PCR-negative
N	17	78
Interstitial lung diseases		
Organizing pneumonia, n (%)	4 (24)	11 (14)
Interstitial pneumonia*, n (%)	2 (12)	11 (14)
Eosinophilic pneumonia, n (%)	2 (12)	1 (1)
Drug-induced lung injury, n (%)	1 (6)	17 (22)
Pulmonary alveolar hemorrhage, n (%)	1 (6)	6 (8)
Infectious disease		
Virus pneumonia, n (%)	3 (18)	0
Bacterial pneumonia, n (%)	2 (12)	6 (8)
Cytomegalovirus pneumonia, n (%)	1 (6)	3 (4)
Mycosis, n (%)	1 (6)	3 (4)
Pneumocystis pneumonia, n (%)	0	17† (22)
Mycobacteriosis, n (%)	0	3 (4)

*exacerbation of interstitial lung disease or acute interstitial pneumonia, †4 of 17 were related to human immunodeficiency virus infection

Table 3 shows the results of BAL cell fraction and peripheral blood eosinophils categorized into the PCR-positive and the PCR-negative groups. BAL eosinophil fraction was higher in the PCR-positive group than in the PCR-negative group; however, only when mildly elevated (> 1% and > 3%). There was no difference in eosinophils in the peripheral blood. The results of the multivariate analysis of the factors involved in increased BAL eosinophil fraction are shown in Table 4. The results showed that PCR positivity of BAL and elevated peripheral blood eosinophils were associated with an eosinophil fraction > 1% and > 3% of BAL. On the other hand, for BAL eosinophil fraction > 5%, only elevated peripheral blood eosinophils were shown to be associated factors. Systemic steroid use decreased the likelihood of BAL eosinophil fraction > 1%.

**Table 3:** Comparison of eosinophils in bronchoalveolar lavage fluid and blood between the PCR-positive group and the PCR-negative group

	PCR-positive	PCR-negative	*p*-value
N	17	78	
Bronchoalveolar lavage fluid			
Total cell count, median (range) ×10^5^	1.26 (0.56–110)	1.31 (0.07-33) (n = 75)	0.798
Macrophage fraction, median (range) %	68.3 (1.8–99.1)	56.6 (6-99.4)	0.240
Lymphocyte fraction, median (range) %	3.9 (0.1–47.5)	24.1 (0.3–73.2)	0.004
Neutrocyte fraction, median (range) %	4.1 (0-63.1)	4.0 (0-92.1)	0.926
Eosinophil fraction, median (range) %	1.4 (0-97.8)	0.4 (0-36.5)	0.030
Eosinophil fraction > 1%, n (%)	9 (53)	19 (24)	0.019
>3%, n (%)	6 (35)	8 (10)	0.017
>5%, n (%)	4 (24)	6 (8)	0.075
Peripheral blood			
White blood cell, median (range) ×10^3^ /m^3^	7.7 (0.1–252)	7.6 (0.1–522)	0.808
Eosinophil fraction, median (range) %	3.3 (0–70)	2 (0-23.5)	0.161
Eosinophil count, median (range) /m^3^	399 (0-17640)	135 (0-2209)	0.213
Eosinophils count > 500/m^3^, n (%)	5 (29)	12 (15)	0.154

Differences among laboratory test results were compared between polymerase chain reaction-positive and -negative cases using the chi-square and Mann–Whitney U tests.

**Table 4:** The factor for elevation of eosinophil fraction in bronchoalveolar lavage

	Odd ratio	95% confidence interval	p-value
BAL eosinophil fraction > 1% *n = 28*			
PCR-positive in BAL	3.8	1.1–14	0.037
Eosinophils in blood > 500/µL	4.6	1.4–15	0.011
User of systemic corticosteroid	0.069	0.008–0.61	0.016
BAL eosinophil fraction > 3% *n = 14*			
PCR-positive in BAL	4.4	1.1–18	0.038
Eosinophils in blood > 500/µL	10	2.7–38	0.001
BAL eosinophil fraction > 5% *n = 10*			
Eosinophils in blood > 500/µL	18	3.9–79	< 0.001

The stepwise logistic regression analysis (with variable increase method) was conducted. The independent variables were PCR-positive in BAL, user of systemic corticosteroid, user of inhaled corticosteroid, bronchial asthma, history of allergies or allergic reactions, and eosinophils in blood > 500/µL.

Of these, nine cases with elevated BAL eosinophil fraction are shown (Table 5). Parainfluenza virus infection was the most common, and two patients diagnosed with eosinophilic pneumonia had parainfluenza infections. Also, two cases of viral pneumonia were included: respiratory syncytial virus and rhinovirus.

**Table 5:** PCR-positive cases with elevated BAL eosinophil fraction

Case	Detected virus	Final diagnosis	Backgrounds	Blood eosinophils (/µL)	BAL eosinophil fraction (%)
1	Parainfluenza virus type 1	Pulmonary aspergillosis	Interstitial pneumonia, ulcerative colitis treated with immunosuppressant	870	4.1
2	Parainfluenza virus type 1	Eosinophilic pneumonia	Bronchial asthma	17,640	97.8
3	Parainfluenza virus type 3	Eosinophilic pneumonia	Bronchial asthma	5522	41.6
4	Parainfluenza virus type 3	Exacerbation of interstitial pneumonia	Rheumatoid arthritis treated with immunosuppressant, diabetes mellitus	484	6.5
5	Parainfluenza virus type 3	Organizing pneumonia	None	445	3.0
6	Parainfluenza virus type 4	Organizing pneumonia	Chronic obstructive pulmonary disease	433	3.1
7	Respiratory syncytial virus type B	Viral pneumonia	Hematological cancer treated with anti-cancer drug	2	1.4
8	Rhinovirus	Viral pneumonia	Human immunodeficiency virus infection, diabetes mellitus	399	2.8
9	Enterovirus	Drug-induced lung injury	Solid cancer treated with anti-cancer drug	58	5.9

Abbreviations: BAL; bronchoalveolar lavage

Elevated BAL eosinophil fraction was defined as > 1%. None of the patients were treated with antiviral therapy.

## Discussion

Several studies have been conducted on eosinophilic airway inflammation caused by respiratory virus infections. In basic research, respiratory syncytial virus infection is often reported to be involved in eosinophilic airway inflammation [4–6]. Clinical studies have demonstrated eosinophilic inflammation due to respiratory virus infections using upper respiratory tract specimens, such as nasal secretions [7, 8]; however, clinical studies using lower respiratory tract specimens, such as BAL, are rare. There have been small studies on rhinovirus and respiratory syncytial virus, showing a slight elevation of BAL eosinophil fraction due to infection [9, 10]. Recent studies have shown that BAL eosinophil fraction is elevated in coronavirus disease 2019 (COVID-19) [11]. Table 6 shows these and other studies showing bronchoalveolar lavage cell analysis in respiratory virus infection subjects [9–19]. Although some studies have shown that respiratory virus infection increases BAL eosinophil fraction, they have been conducted in patients with allergic diseases such as bronchial asthma, and the increase in the eosinophil fraction was slight, as in the present study. On the other hand, two studies in the clinical setting, like the present study, were conducted in patients with underlying pulmonary diseases; however, they did not find an increased BAL eosinophil fraction due to viral infection. Unlike the present study, none of these studies had as their main objective the analysis of BAL eosinophil fraction.

**Table 6:** Articles showing bronchoalveolar lavage cell analysis in subjects infected with respiratory viruses

Year	Country	Study design	Population	Subjects	BAL eosinophil elevation, Y/N	Virus species
1994	US	Prospective	Adults	Allergic rhinitis (n = 7), control (n = 5)	Y	Rhinovirus*
2000	Korea	Prospective	Children	Acute asthma (n = 18), RS virus bronchiolitis (n = 20), control (n = 14)	N	RS virus
2003	Korea	Prospective	Children	Acute asthma (n = 18),.RS virus bronchiolitis (n = 22), control (n = 14)	N	RS virus
2005	Korea	Prospective	Children	Acute asthma (n = 16), RS virus bronchiolitis (n = 18), control (n = 14)	N	RS virus
2008	UK	Prospective	Adults	Atopic asthma (n = 11), control (n = 17)	Y	Rhinovirus*
2013	Italy	Cross-sectional study	Children	Recurrent/chronic lower respiratory disorders with virus positive (n = 50), negative (n = 30)	N	Multiple
2013	Netherlands	Prospective	Adults	Atopic asthma (n = 13), control (n = 11)	N	Rhinovirus*
2014	Netherlands	Prospective	Adults	Allergic asthma (n = 14), control (n = 14 )	Y	Rhinovirus*
2019	Japan	Retrospective	Adults	Interstitial lung diseases with respiratory virus infection (n = 50), without infection (n = 56)	N	Multiple
2021	Morocco	Prospective	Adults	Severe COVID-19 (n = 45), control (n = 25)	Y	SARS-CoV-2
2022	UK	Prospective	Adults	Bronchial asthma (n = 11), control (n = 12)	Y	Rhinovirus*

Abbreviations: BAL; bronchoalveolar lavage, COVID-19; coronavirus disease 2019, SARS-CoV-2; severe acute respiratory syndrome coronavirus 2, *experimental infection of rhinovirus 16

Eosinophilic airway inflammation caused by respiratory virus infections is considered a protective response to respiratory viruses, and basic medical and clinical studies have shown that eosinophilic inflammation is protective against respiratory viral infections [20–23]. Eosinophils produce and contain molecules with antiviral activity, such as RNase and active nitrogen species [22]. In addition, eosinophils can capture and inactivate viruses [23]. Therefore, increased BAL eosinophil fraction due to respiratory virus infection is a rational response to protect against respiratory viruses.

In the present study, two patients with positive results for parainfluenza virus had a marked elevation of BAL eosinophil fraction and were diagnosed with eosinophilic pneumonia (Case 2 and 3). They had a history of bronchial asthma and had uncontrolled asthma symptoms prior to the examination. Chest imaging revealed a non-regional subpleural infiltrate in the right lower lobe in Case 2, and a typical finding of chronic eosinophilic pneumonia, a photographic negative of pulmonary edema, in Case 3. After bronchoscopy, the patient was diagnosed with eosinophilic pneumonia and started on systemic steroid therapy. Clinical and imaging findings improved quickly, and tapering was completed in about 2 months. Their clinical course was consistent with the usual course of chronic eosinophilic pneumonia. In these two patients, eosinophilic pneumonia was probably caused by parainfluenza infection since there was no other obvious cause. Although there have been reports of cases of eosinophilic pneumonia with a marked elevation of BAL eosinophil fraction in influenza virus infection and COVID-19 [24–26], there are no reports of eosinophilic pneumonia caused by parainfluenza virus. There has been a case report of parainfluenza virus pneumonia; however, the increase of BAL eosinophils was mild and not eosinophilic pneumonia [27]. It is likely that there are many more cases of eosinophilic pneumonia caused by respiratory viral infections in clinical practice. Highly sensitive multiplex testing will further clarify the relationship between respiratory viral infections and allergic respiratory diseases.

There were some limitations to this study. First, this was a retrospective single-center study. PCR testing was performed as a routine test on BAL specimens during the study period. However, it was not mandatory and may not have been performed on cases deemed unnecessary by the attending physician. The number of such cases is unknown. It is possible that there was a bias in the backgrounds of the cases included in this study or in the respiratory viruses prevalent in our region. Second, the number of positive cases was too small to properly perform a multivariate analysis of all possible confounders. The statistical analysis on BAL eosinophil fraction should have been performed with the eosinophil fraction measured as a continuous variable; however, we had to replace it with a binary variable to reduce the effect of outliers due to the small population. Third, the degree of eosinophilic airway inflammation is different for different respiratory viruses. A previous report showed that respiratory syncytial virus infection caused higher eosinophil counts than metapneumovirus infection in peripheral blood [28]. When comparing rhinovirus and influenza virus, infection of the former was higher in peripheral blood [29]. Additionally, some respiratory viruses, adenovirus, and bocavirus, probably do not cause eosinophilic inflammation [30, 31]. Further studies on each respiratory virus species are necessary. Finally, patient bias and factors other than respiratory virus infection may influence an increase in BAL eosinophil fraction. A history of bronchial asthma tended to be more common in the PCR-positive group. Even though multivariate analysis showed no association with elevated BAL eosinophil fraction, it may have influenced the results. Alternatively, this background bias may have been inevitable, as respiratory virus infections are reported to be more likely to occur in patients with bronchial asthma [32]. There were more cases of drug-induced lung injury and pneumocystis pneumonia not related to human immunodeficiency virus infection in the PCR-negative group. Since both diseases elevate BAL lymphocyte fraction, it is likely that BAL fraction of the PCR-negative group tended to be higher [3, 33]. As a result, BAL eosinophil fraction of the PCR-negative group may have been relatively lower.

## Conclusion

In this study, we found that BAL eosinophil fraction was increased in patients infected with respiratory viruses in the lower respiratory tract. The degree of eosinophilic inflammation caused by respiratory virus infections is mild; however, it can occasionally cause eosinophilic pneumonia. Clinicians should also consider respiratory virus infection as a differential cause of elevated BAL eosinophils.

## Data Availability

The datasets generated and/or analysed during the current study are not publicly available due privacy and ethical restrictions but are available from the corresponding author on reasonable request.

## Abbreviations

BAL: Bronchoalveolar lavage
PCR: polymerase chain reaction
